# How Are BMI, Nutrition, and Physical Exercise Related? An Application of Ordinal Logistic Regression

**DOI:** 10.3390/life12122098

**Published:** 2022-12-14

**Authors:** Hongwei Wang, Fernando G. Quintana, Yunlong Lu, Muhammad Mohebujjaman, Kanon Kamronnaher

**Affiliations:** 1 Department of Mathematics and Physics, Texas A&M International University, Laredo, TX 78041, USA; 2 Department of Biology and Chemistry, Texas A&M International University, Laredo, TX 78041, USA; 3 School of Mathematics and Statistics, Beihua University, Jilin 132013, China; 4 School of Mathematical and Statistical Sciences, Clemson University, Clemson, SC 29634, USA

**Keywords:** ordinal logistic regression, BMI, health survey, South Texas

## Abstract

Background: This paper performs a detailed ordinal logistic regression study in an evaluation of a survey at a university in South Texas, USA. We show that, for categorical data in our case, ordinal logistic regression works well. Methods: The survey was designed according to the guidelines in diet and lifestyle from the American Heart Association and the United States Department of Agriculture and was sent out to all registered students at Texas A&M International University in Laredo, Texas. Data analysis included 601 students’ results from the survey. Data analysis was conducted in Rstudio. Results: The results showed that, compared with students who do not have enough whole grain food and exercise, those who have enough in both tend to have normal BMIs. As age increases, BMI tends to be out of the normal range. Conclusions: Because BMI in this research has three categories, applying an ordinal logistic regression model to describe the relationship between an ordered categorical response variable and more explanatory variables has several advantages compared with other models, such as the linear regression model.

## 1. Introduction

Obesity in children is connected with factors such as eating habits [1] and has distinct associations with food group intakes, physical activity, and socio-economic status [2]. BMI has been discussed in many previous research studies such as [3,4,5,6] as a survey variable in other statistical analyses such as Bayesian inference, the pseudo maximum likelihood approach, multivariate analysis, and ordinary least squares, respectively. The ordinal logistic regression model (also known as the proportional odds model [7]) is an ordinal regression model, which is a regression model for ordinal dependent variables that allows for more than two ordered response categories. The ordinal logistic regression model has been applied in many areas of public health (e.g., [8] in quality of life studies, [9] in pregnancy outcomes, and [10] in patients diagnosed as lung adenocarcinoma). This study applies ordinal logistic regression analysis to a healthy survey about body mass index (BMI). The survey was designed and sent to all the registered students by email in Fall 2019 at Texas A&M International University (TAMIU), a member of the Texas A&M System, located in Laredo, Texas, on the border of the United States and Mexico. Questions about students’ height, weight, age, daily food, physical activities, and demographic information were included in the survey. The ordinal logistic regression model in this research predicts the BMI categories by daily food intake, duration of physical activities, and age, and interprets the relationship between those variables. How students eat and do physical activities is expected to affect their BMI categories.

This paper is organized as follows: Section 2 presents the background of the survey and details of data analysis. In Section 3, the main results of the ordinal logistic regression are presented. Assumption analysis and interpretation of the model are presented in Section 4. Finally, the importance of this study, why it is new, and the contribution of this work to the scientific community are presented in Section 5.

## 2. Materials and Methods

The relationship between BMI percentiles and kidney function and the probability of fatty liver and hepatomegaly was discussed in [11], and it showed that the obesity rates among Mexican-American children are higher than obesity rates of the general American child population. The U.S. Department of Health and Human Services Office of Minority Health [12] indicated that, among Hispanic-American women, 78.8% are overweight or obese, compared to only 64% of non-Hispanic White women. In 2017, Hispanic high school students were 50% more likely to be obese compared to non-Hispanic white youth. In 2018, Hispanic Americans were 1.2 times more likely to be obese than non-Hispanic Whites. Katherine pointed out that Laredo, Texas, where Hispanics make up 96% of the population, is the least diverse area in the United States [13]. Under this circumstance, it is important and necessary to do research for about college students at TAMIU in Laredo to understand why students are underweight or overweight or obese.

The objective of this research is to predict a student’s BMI category based on factors such as the amount of intake of whole grain food and protein, the extent of physical activity, and age. It is well-known that many whole grains are good or excellent sources of dietary fiber [14]. Dietary fiber from whole grains as part of an overall healthy diet may help improve blood cholesterol levels and lower the risk of heart disease, stroke, obesity, and type 2 diabetes. The American Heart Association’s Diet and Lifestyle Recommendations aim for at least 150 min of moderate physical activity or 75 min of vigorous physical activity—or an equal combination of both—each week [15].

Based on the information above, a survey was designed and includes questions about diet, physical activities, and demographic information. The first 11 questions ask about whether or not the students eat fruit, green vegetables, orange vegetables, whole grain food, protein food and how much they eat in a week. Questions 12–15 ask about physical activities, including cardio, strength training, stretching, and relaxation. The last part asks about demographic information, such as age, weight, height, race, education level of parents, marital status, and residence (whether living in the student dorms or living at home). According to the answers from the survey, this research defines variables “wholegrain”, “protein”, “exercise”, and “age”. The variable “wholegrain” is the total amount of wholegrain food students consume in a week, and the value is between 0 to 49, for which every unit stands for 1 ounce raw weight. Similarly, the variable “protein” is the amount of protein food, including animal protein and plant protein, students consume in a week, and it is between 0 to 64, for which every unit stands for 1 ounce. Students are also asked how many days in a week they do at least 30 min of strength training, cardio training, or stretching and relaxation. The variable “exercise” is defined as the sum of days that students do physical activities in a week. It ranges between 0 to 21, and every unit represents at least 30 min. The height and weight provided by students in the survey BMI can be calculated as follows [16,17]:

(1) $$\mathrm{BMI}=\frac{{\mathrm{mass}}_{kg}}{{\mathrm{height}}_{m}^{2}}=703\times \frac{{\mathrm{mass}}_{lb}}{{\mathrm{height}}_{in}^{2}}.$$

Based on the numerical value of BMI, it can be defined in four categories: underweight with BMI below 18.5 (category 1), normal or healthy weight with BMI between 18.5 and 24.9 (category 2), overweight with BMI above 24.9 but less than 29.9, and obese with 29.9 and above [16,18,19,20]. Overweight and obese are combined into one category (category 3).

Out of 601 students, there are 255 in normal BMI category, 316 are in the overweight or obese BMI category, and the rest (30) are the underweight BMI category. The summary of the data set in this research is described in Table 1.

**Figure 1:**
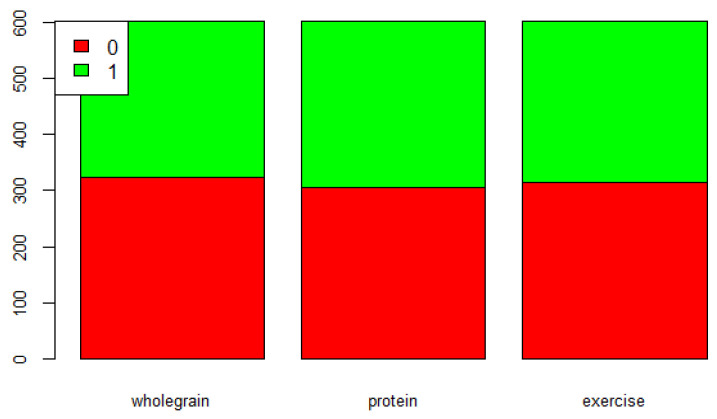
Distribution of wholegrain, protein, and exercise.

We calculate the mean values of “wholegrain”, “protein”, and “exercise”. The binary variable “wholegrainenough” is defined as 1 if the value of “wholegrain” is greater than or equal to the mean value; otherwise, “wholegrainenough” is defined as 0. The binary variables “proteinenough” and “exerciseenough”are defined the same way. The distribution of “wholegrainenough”, “proteinenough”, and “exerciseenough” are shown in Figure 1. The red color represents a binary value of 0 while the green color represents 1. The *y*-axis represents the number of observations, and the *x*-axis represents three different variables (“wholegrain”, “protein”, and “exercise”). Figure 1 shows that there are more students who have wholegrain and exercise amounts below the mean values than students who have both amounts above the mean values.

Michael Foley summarized the ordinal logistic regression model in his data science notes [21] as follows:

(2) $$logit\left(P(Y\leq j)\right)=log\left(\frac{P(Y\leq j)}{P(Y>j)}\right)=\alpha_{j}-\beta X,$$

where j=1,2,…,J−1 are the levels of the ordinal outcome BMI category variable *Y*, *J* is an integer that represents the number of categories of *Y*, $\alpha_{j}$s are J−1 intercepts, and β is the slope for all predictors. Note that the proportional odds model assumes that there is one common slope parameter for all predictors.

In this paper, J=3 and the BMI category Y=1, 2, and 3. Then, P(Y≤j) is the cumulative probability of *Y* less than or equal to a specific category *j*. Note that P(Y≤3)=1. The odds of being less than or equal to a particular category can be defined as

$$\frac{P(Y\leq j)}{P(Y>j)},\;j=1,2.$$

Since P(Y>3)=0 and because of zero division, the case j=3 is avoided. The logodds is also known as the logit, so that

(3) $$log\left(\frac{P(Y\leq j)}{P(Y>j)}\right)=logit\left(P(Y\leq j)\right).$$

An odds ratio (OR) is a measure of association between an exposure and an outcome. In a logistic regression, the regression coefficient is the estimated increase in the logodds of the outcome per unit increase in the value of the exposure [22]. The ordinal logistic regression model can be defined as [23]

(4) $$logit\left(P(Y\leq j)\right)=\beta_{j0}+\beta_{j1}x_{1}+...+\beta_{jp}x_{p},$$

for j=1,…,J−1, where *p* is the number of predictors, $x_{1},x_{2},\ldots ,x_{p}$ are predictors variables, and $\beta_{j}$ is the regression coefficient for the predictor variable $x_{j}$. Due to the parallel lines assumption, the intercepts are different for each category, but the slopes are constant across categories, which reduces Equation (4) to

(5) $$logit\left(P\left(Y\leq j\right)\right)=\beta_{j0}+\beta_{1}x_{1}+...+\beta_{p}x_{p},$$

where we define $\beta_{1}:=\beta_{j1},\beta_{2}:=\beta_{j2},\cdots ,\beta_{p}:=\beta_{jp}$, for j=1,⋯,J−1. Bruin [23] pointed out that, in R, the ordinal logistic regression model is summarized as

(6) $$logit\left(P\left(Y\leq j\right)\right)=\beta_{j0}-\eta_{1}x_{1}-\ldots -\eta_{p}x_{p},$$

where $\eta_{i}=-\beta_{i}.$

An ordinal logistic regression model predicting BMI categories by “wholegrainenough”, “proteinenough”, “exerciseenough” and “age”, was built in Rstudio, and the necessary data analysis was performed. The packages foreign, reshape2, ggplot2, Hmisc, MASS, and caret were used. We use the polr function to build the ordinal logistic regression. Clean data were saved in the data set southtexas_complete, and BMI was defined by the variable BMIcode.f.

## 3. Results

The results of the ordinal logistic regression model are shown in Table 2.

The estimated models are written as:

(7) $$logit(\hat{p}\left(Y\leq 1\right))=0.39-\left(-0.34\right)*\mathrm{we}-\left(0.01\right)*\mathrm{pe}-\left(-0.48\right)*\mathrm{ee}-\left(0.05\right)*\mathrm{age},$$

and

(8) $$logit(\hat{p}\left(Y\leq 2\right))=3.75-\left(-0.34\right)*\mathrm{we}-\left(0.01\right)*\mathrm{pe}-\left(-0.48\right)*\mathrm{ee}-\left(0.05\right)*\mathrm{age},$$

where $\hat{p}$ is the estimated probability. Note that we abbreviate “wholegrainenough” as “we”, “proteinenough ” as “pe”, and “exerciseenough ” as “ee” in the above models. The results of odds ratios are shown in Table 3. We observe that the amount of intake of whole grain and protein, amount of physical exercise, and age are significantly related to the BMI categories.

## 4. Discussion

Models with terms that reflect ordinal characteristics such as monotone trend have improved model parsimony and power [7]. This makes the ordinal logistic regression model important. This research does not consider ordinary least square analysis because BMI category in this study is a non-interval outcome variable, which violates the assumptions of ordinary least square [24]. ANOVA would be a good option if there is only one continuous predictor [25]. Multinomial logistic regression [26] works similarly to ordinal logistic regression except that it is assumed that there is no order to the categories of the outcome variable (i.e., categories are nominal). In this research, BMI has three categories: category 1 is underweight, category 2 is normal, and category 3 is overweight or obese. BMI category in this study is ordered, and the distances between different categories are not consistent because the distance between underweight and normal is shorter than the distance between normal to overweight or obese. Thus, the ordinal logistic regression model can be applied in this case.

With the models above, we interpret that, for a one unit increase in “wholegrainenough” (from having less than the average amount of whole grain food consumed by students at TAMIU to the average or above average amount), a (−0.34) increase (or a 0.34 decrease) in expected BMI on the logodds measurement is expected, if we hold other variables in the model constant. For a one-unit increase in “proteinenough” (from having less than the average amount of protein consumed by students at TAMIU to the average or above average amount), we expect a 0.01 increase in expected BMI on the logodds measurement, if we hold other variables in the model constant. Similarly, we expect a (−0.48) increase (or a 0.48 decrease) and a (0.05) increase in expected BMI on the logodds measurement, respectively, if we hold other variables in the model constant for every one unit increase in “exerciseenough” (from having less than the average amount of exercise time by students at TAMIU to the average or above average amount) and “age”.

We can also analyze the odds ratios as displayed in Table 3. A 95% confidence interval of the odds ratios is constructed and displayed in the last two columns. For students who have more than the average intake of whole grain food, the odds of being more likely to have in the category BMI overweight or obese or the category underweight versus normal is multiplied 0.72× (i.e., decreases 28%), if we hold other variables in the model constant. For students who have more than the average intake of protein, the odds of being more likely to have in the category BMI overweight or obese or the category underweight versus normal is multiplied 1.01× (i.e., increases 1%), if we hold other variables in the model constant. For students who have more than the average time in exercise, the odds of being more likely to have in the category BMI overweight or obese or the category underweight versus normal is multiplied 0.62× (i.e., decreases 38%), if we hold other variables in the model constant. For every one-unit increase in student’s age, the odds of being more likely to have in the category BMI overweight or obese or the category underweight versus normal is timed 1.05× (i.e., increases 5%), if we hold other variables in the model constant. Compared with results from previous studies [27,28,29] that diets in low carbohydrate and high protein benefit weight control, the results from this research show that consumption of whole grain food can actually help keep BMI in the normal range and too much protein intake is associated with abnormal BMI. These results will guide students at TAMIU in improving overall health and food service in the cafeteria on campus to better serve students.

### 4.1. Proportional Odds Assumption

Ordinal logistic regression assumes that the relationship between each pair of outcome groups is the same, i.e., ordinal logistic regression assumes that the coefficients that describe the relationship between any two categories of the dependent variable are the same. Thus, to assess the quality of our model, we would like to check whether or not the proportional odds assumption is sustainable.

Based on the analysis in [23], for individual logistic regressions, we will graph predicted logits with a single predictor; the outcome groups are described by different categories of response variables (BMI category ≥2 and BMI category ≥3). If there is no difference between predicted logits for different levels of a predictor, such as “wholegrainenough”, no matter if the outcome is BMI category ≥2 or BMI category ≥3, then it is valid to conclude that the proportional odds assumption is sustainable (i.e., if the difference between logit for “wholegrainenough=0” and “wholegrainenough=1” is the same while the result is BMI category ≥2 as the difference while the result is BMI category ≥3; then, the proportional odds assumption is sustainable).

When we regressed the response variable on independent variables one at a time, if the proportional odds assumptions is not sustainable, the results we would obtain are displayed in Table 4. We define a function that calculates the logodds of being no less than each value of the target variable. For the purpose of this research, the logodds of BMI category being at least 2 and at least 3 should be analyzed. Because the dependent variable has three levels, there are columns Y≥1, Y≥2, and Y≥3. Inside the defined function, there is another function, a transformation function, transforming a probability to a logit. Therefore, we feed probabilities of the category of BMI being greater than 2 or 3 to the transformation function, and it returns the logit transformations of these probabilities. For example, in the column Y≥2, the category of BMI ≥2 will be evaluated to a vector with values FALSE and TRUE, and readers will obtain the proportional of probability that BMI category ≥2 by taking the mean of the FALSE/TRUE vector. In the column Y≥1, all entries are “*∞*” because all the data discussed in this article fall within the BMI category ≥1.

We ran several binary logistic regressions with different cut-points on the dependent variables, and checked the equality of coefficients across cut-points. This helped us evaluate the proportional odds assumption. A model was also built to estimate the effect of intake enough whole grain food (i.e., the variable “wholegrainenough”) on choosing “BMI normal” versus “BMI overweight or obese” or “BMI underweight”(results are shown in Table 5). Similarly, the effect of taking in enough whole grain food on choosing “BMI normal” or “BMI overweight or obese” versus “BMI underweight” was estimated (results are shown in Table 6). In Table 5, the intercept for this model (0.51) matches the predicted value (shown in Table 4) in the cell for “wholegrainenough” equal to “No” in the column for Y≥2; “wholegraineough” equals “yes” is the sum of the intercept and the coefficient for “wholegrainenough” (i.e., 0.51+(−0.44)=0.07) in Table 5. In Table 6, the intercept for this model (−2.89) is the same as the predicted value (shown in Table 4) in the row of “wholegrainenough” equal to “No” and the column of Y≥3; in the row of “wholegraineough” equals “Yes”and the column of Y≥3, the entry is equal to the intercept plus the coefficient for “wholegrainenough” (i.e., −2.89+(−0.12)=−3.01) in Table 6. Thus, this suggests that the proportional odds assumption does hold for the predictor variable “wholegrainenough”. Table 4 is reproduced to obtain Table 7 by taking the difference between the last two columns, and setting the third column to zero.

**Figure 2:**
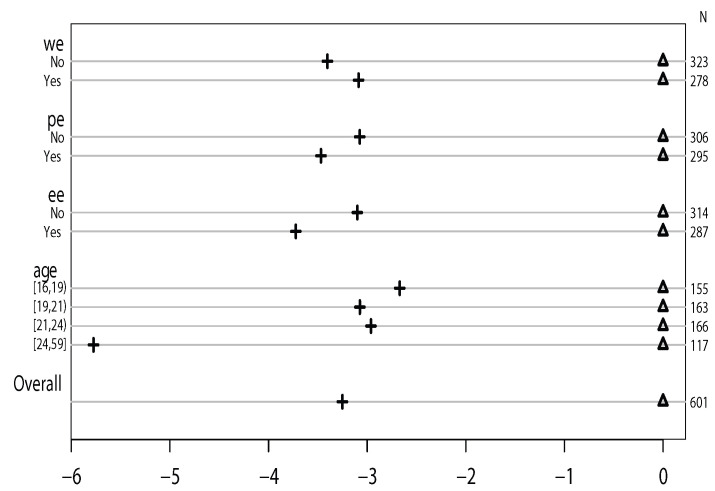
Proportional odds assumption.

In the results, for example, when “wholegrainenough” equals “No”, the difference between the predicted value for Y≥3 and Y≥2 is −3.40 (=−2.89−0.51 in Table 4). For “wholegrainenough” equals “Yes”, the difference between the predicted value for Y≥3 and Y≥2 is −3.09 (≈−3.01−0.07 in Table 4). The difference between −3.09 and −3.40 is acceptable. The values for “exerciseenough” equaling “No” and “Yes” are −3.08 and −3.47, respectively. The values for “proteinenough” equaling “No” and “Yes” are −3.10 and −3.72, respectively. These suggest that the parallel slopes’ assumption holds. The differences are shown in Figure 2. Thus, we conclude that the effects of whether or not having enough whole grain food, protein, and exercise time are the same for the transitions from “BMI normal” to “BMI overweight or obese” and “BMI overweight or obese” to “BMI underweight” (i.e., the proportional odds assumption of our model holds).

Figure 2 shows whether or not the proportional assumption holds. If it does, for each independent variable, the distance between the symbols for each set of categories of the dependent variable should stay similar [23]. To display this, Figure 2 is constructed by taking the differences of Y≥2 and Y≥3 and normalizing all the first sets of coefficients to zero to make it a common reference point. The *x*-axis is for the value of logit and the *y*-axis is for the different categories of each predictor variable. Because “age” is a continuous variable, it is distributed equally into four intervals. Δ is the common reference point, and + is the location of each category. In Figure 2, the distances between the two sets of coefficients (“No” and “Yes”) of the variables “wholegrainenough”, “proteinenough”, and “exerciseenough” are similar. Thus, the proportional assumption holds in this research.

### 4.2. Predicted Probability

Once the assessment of whether or not the proportional odds assumptions of the model holds is completed, predicted probabilities can be obtained. We change “age ” for different values (0 and 1) of “wholegrainenough”, “proteinenough”, and “exerciseenough” and evaluate the probabilities of being in each category of BMI. The first six rows of results are displayed in Table 8. One of the advantages of ordinal logistic regression is the prediction we can have from the model, which means that one value of a predictor variable is associated with the change of value in response variable. The first two rows in Table 8 show that, if the “wholegrainenough”is changed from level 0 to level 1, for the same age subject, the probability of having normal BMI will be increased from 0.40 to 0.48, while the probability of having overweight or obese will be decreased from 0.55 to 0.49. A similar study from other researchers such as [30] would not be able to have prediction as we did in this study.

**Figure 3:**
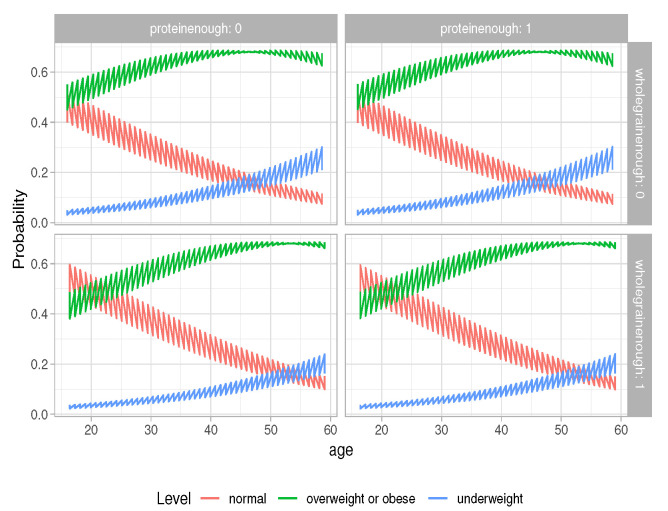
Predicted probability “wholegrainenough” and “proteinenough”.

**Figure 4:**
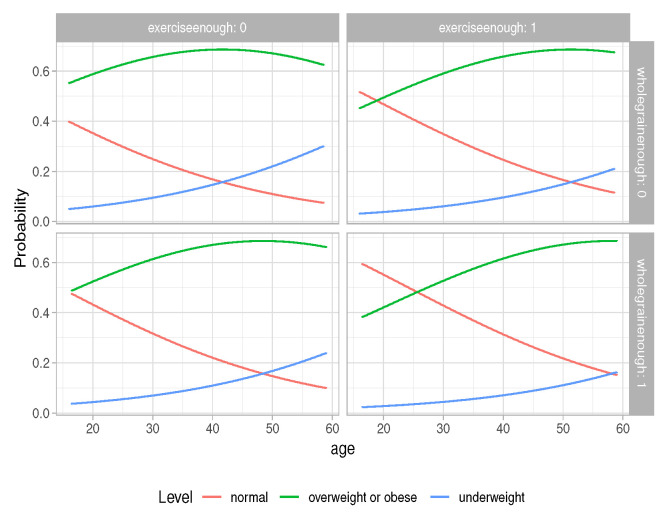
Predicted probability “wholegrainenough” and “exerciseenough”.

We also plot the predicted probabilities; they are described using a line, colored by level of BMI, and facetted by levels of “wholegrainenough” and “proteinenough”, which are shown in Figure 3, and by levels of “wholegrainenough” and “exerciseenough”, which are shown in Figure 4. In both Figure 3 and Figure 4, it is found that, as age increases, the probability of having BMI normal decreases. If having protein over the average amount or having exercise time over the average amount is not taken into consideration, at the same age, the probability of having BMI normal is larger for students who have an above average amount of whole grain food than for the students who have less than the average amount of whole grain food.

Compared with the research which only studies the relationship between eating habits and BMI [5], this research is significant not only because this is one of the very few research studies which uses an ordinal logistic regression model to analyze BMI, but also because this research studies the relationships between BMI, eating habit, and physical activities. This might help readers better understand how BMI is impacted by different factors in daily life. In addition, this is one of the very few research studies about public health in college students in South Texas. Therefore, this research has significant importance to the Hispanic population. The results will help the population in this area better understand how to eat and exercise, and improve their health and well-being. Most importantly, this research comes out with a new result in BMI. Eating well and doing enough workouts help people avoid being overweight or obese [5,31], which has been known for years. This research confirms the previous results but also points out that eating well and having enough workouts can help people avoid being underweight, which is as unhealthy as being overweight or obese.

## 5. Conclusions

For students who have more than the average intake of whole grain food, BMI is more likely to be normal (the odds of being more likely to have BMI overweight or obese or underweight versus normal decreases 29%), holding constant all other variables. For students who have more than the average intake of protein, BMI is more likely to be out of normal range (the odds of being more likely to have BMI overweight or obese or underweight versus normal increases 1%), if we hold all other variables constant. This is because Mexican food is rich in protein (in the form of animal or plant protein or both) compared to other diets. Therefore, the average amount of protein intake might be above the amount recommended by [32] for the general population. For students who have more than the average time in exercise, BMI tends to be normal (the odds of being more likely to have BMI overweight or obese or underweight versus normal decreases 38%), if we hold all other variables constant. For every one unit increase in student’s age, BMI is more likely to be out of normal range (the odds of being more likely to have BMI overweight or obese or underweight versus normal increases 5%), holding constant all other variables. This confirms that failing to have enough whole grain food and exercise likely causes students in South Texas to be overweight as well as underweight.

Obese individuals should exercise consistently to achieve significant improvements in their health. This has been pointed out in [31]. An inverse relationship between whole grain food intake and BMI has been presented in [33]. This article not only confirms the conclusion of previous research studies [31,33] but also points out that BMI underweight is related to an unhealthy diet and inadequate physical activities. Either BMI underweight or BMI overweight or obese is not healthy, which should attract the attention of people in South Texas. This result is new and important for improving the overall health of the Hispanic population.

Because BMI in this research has three categories, applying an ordinal logistic regression model to describe the relationship between an ordered categorical response variable and more explanatory variables has several advantages compared to other models, such as the linear regression model. Fagerland summarized in [34] that the advantages of ordinal logistic regression include mathematical flexibility and ease of use, the exponential form of the regression coefficients, ability to be interpreted as odds ratios, and the possibility of several different logistic models, which can be found in this work.

### Suggestions for Further Study

Ordinal logistic regression has a very strict model assumption of parallel lines (i.e., different logistic models have the same coefficients) for all response variable categories. One can try the multinomial logistic regression model [26] as an alternative analysis if the parallel lines assumption fails. In addition, the sleeping pattern should be taken into consideration if possible to analyze the impacts on BMI.

## Figures and Tables

**Table 1:** Summary of data set.

	Min.	1st Qu.	Median	Mean	3rd Qu.	Max.
wholegrain	1.0	6.0	14.0	15.9	24.0	49.0
protein	1.0	18.0	30.0	31.8	42.0	64.0
exercise	2.0	3.0	6.0	6.6	9.0	16.0
age	16.0	18.0	20.0	21.8	23.0	59.0
BMI category						
Normal		Overweight or obese			Underweight	
255		316			30	

**Table 2:** Ordinal logistic regression model.

	Value	Std. Error	*t* Value	*p* Value
		Coefficients		
wholegrainenough	−0.34	0.17	−1.95	0.05
proteinenough	0.01	0.17	0.05	0.96
exerciseenough	−0.48	0.17	−2.86	≤0.01
age	0.05	0.02	3.37	≤0.01
		Intercepts		
normal|overweight or obese	0.39	0.35	1.12	0.26
overweight or obese|underweight	3.75	0.41	9.26	≤0.01
		Residual Deviance: 995.54		
		AIC: 1007.54		

**Table 3:** Odds ratio and confidence intervals.

Variables	OR	2.5%	97.5%
wholegrainenough	0.72	0.51	1.00
proteinenough	1.01	0.73	1.40
exerciseenough	0.62	0.45	0.86
age	1.05	1.02	1.08

**Table 4:** Linear predicted values without proportional odds assumption.

Variables	Categories	N	Y≥1	Y≥2	Y≥3
wholegrainenough	No	323	*∞*	0.51	−2.89
	Yes	278	*∞*	0.07	−3.01
proteinenough	No	306	*∞*	0.30	−2.77
	Yes	295	*∞*	0.31	−3.16
exerciseenough	No	314	*∞*	0.56	−2.54
	Yes	287	*∞*	0.03	−3.69
age	[16,19)	155	*∞*	−0.12	−2.79
	[19,21)	163	*∞*	0.23	−2.84
	[21,24)	166	*∞*	0.32	−2.65
	[24,59)	117	*∞*	1.02	−4.75
Overall		601	*∞*	0.31	−2.95

**Table 5:** Effect of enough intake of whole grain food on choosing “normal” versus “overweight or obese” or “underweight”.

Intercept	0.51
wholegrainenough coefficient	−0.44

**Table 6:** Effect of enough intake of whole grain food on choosing “normal” or “overweight or obese” versus “underweight”.

Intercept	−2.89
wholegrainenough coefficient	−0.12

**Table 7:** Reproduced linear predicted values without proportional odds assumption.

Variables	Categories	N	Y≥1	Y≥2	Y≥3
wholegrainenough	No	323	*∞*	0	−3.40
	Yes	278	*∞*	0	−3.09
proteinenough	No	306	*∞*	0	−3.08
	Yes	295	*∞*	0	−3.47
exerciseenough	No	314	*∞*	0	−3.10
	Yes	287	*∞*	0	−3.72
age	[16,19)	155	*∞*	0	−2.67
	[19,21)	163	*∞*	0	−3.07
	[21,24)	166	*∞*	0	−2.96
	[24,59)	117	*∞*	0	−5.77
Overall		601	*∞*	0	−3.25

**Table 8:** Predicted probability.

N	we	pe	ee	Age	Normal	Overweight or Obese	Underweight
1	0	0	0	16.00	0.40	0.55	0.05
2	1	0	0	16.62	0.48	0.49	0.04
3	0	0	0	17.23	0.39	0.56	0.05
4	1	0	0	17.85	0.46	0.50	0.04
5	0	0	0	18.47	0.37	0.57	0.06
6	1	0	0	19.08	0.45	0.51	0.04

## Data Availability

Data supporting reported results can be found from the authors if request.
